# A new method to detect event-related potentials based on Pearson’s correlation

**DOI:** 10.1186/s13637-016-0043-z

**Published:** 2016-06-07

**Authors:** William Giroldini, Luciano Pederzoli, Marco Bilucaglia, Simone Melloni, Patrizio Tressoldi

**Affiliations:** 1 EvanLab, Via dei Ricci, 22 - 50023 Impruneta, 50023 Florence, Italy; 2 Dipartimento di Psicologia Generale, Università di Padova, via Venezia, 8, 35131 Padova, Italy

**Keywords:** Event-related potentials, Brain-computer interfaces, Pearson’s correlation

## Abstract

Event-related potentials (ERPs) are widely used in brain-computer interface applications and in neuroscience.  Normal EEG activity is rich in background noise, and therefore, in order to detect ERPs, it is usually necessary to take the average from multiple trials to reduce the effects of this noise.  The noise produced by EEG activity itself is not correlated with the ERP waveform and so, by calculating the average, the noise is decreased by a factor inversely proportional to the square root of *N*, where *N* is the number of averaged epochs. This is the easiest strategy currently used to detect ERPs, which is based on calculating the average of all ERP’s waveform, these waveforms being time- and phase-locked.  In this paper, a new method called GW6 is proposed, which calculates the ERP using a mathematical method based only on Pearson’s correlation. The result is a graph with the same time resolution as the classical ERP and which shows only positive peaks representing the increase—in consonance with the stimuli—in EEG signal correlation over all channels.  This new method is also useful for selectively identifying and highlighting some hidden components of the ERP response that are not phase-locked, and that are usually hidden in the standard and simple method based on the averaging of all the epochs.  These hidden components seem to be caused by variations (between each successive stimulus) of the ERP’s inherent phase latency period (jitter), although the same stimulus across all EEG channels produces a reasonably constant phase. For this reason, this new method could be very helpful to investigate these hidden components of the ERP response and to develop applications for scientific and medical purposes. Moreover, this new method is more resistant to EEG artifacts than the standard calculations of the average and could be very useful in research and neurology.  The method we are proposing can be directly used in the form of a process written in the well-known Matlab programming language and can be easily and quickly written in any other software language.

**Electronic supplementary material:**

The online version of this article (doi:10.1186/s13637-016-0043-z) contains supplementary material, which is available to authorized users.

## Introduction

The event-related potential (ERP) is an electroencephalographic (EEG) signal recorded from multiple brain areas, in response to a single short visual or auditory stimulus or muscle movement [25, 27].

ERPs are widely used in brain-computer interface (BCI) applications and in neurology and psychology for the study of cognitive processes, mental disorders, attention deficit, schizophrenia, autism, etc. [2, 15, 18].

ERPs are weak signals compared to spontaneous EEG activity, with very low signal-to-noise ratio (SNR) [12], and are typically comprised of two to four waves of low amplitude (4–10 μV) with a characteristic positive wave called P300, which has a latency period of about 300 ms in response to the stimulus [17]. The detection of ERPs is an important problem, and several methods exist to distinguish these weak signals. Indeed, ERP analysis has become a major part of brain research today, especially in the design and development of BCIs [26].

In this paper, the definition and description of the ERP is focused mainly on the P300 because it is the simplest way to present our new ERP detection method.

We will not be considering fast evoked potentials (EVP), such as the brainstem auditory EVP, because they require a fast sampling rate (around 1000 Hz), an averaging of perhaps 1000 responses, and an upper frequency filtering of about 100 to 1000 Hz.

Since the ERP is considered a reproducible response to a stimulus, with relatively stable amplitude, waveform and latency, the standard method to extract ERPs is based on the repeated presentation of the stimulus about 100 times, with a random inter-stimulus time of a few seconds. This strategy allows calculating the ERPs by averaging several epochs that are time-locked and phase-locked.

Each epoch is constituted generally by a pre-stimulus, stimulus, and post-stimulus interval.

The averaging method is based on the assumption that the noisy EEG activity is uncorrelated with the ERP waveform, and consequently calculating the average decreases the noise by a factor of 1/*√ N* (inverse of square root of *N*), where *N* is the number of averaged epochs. Since the background EEG activity has a higher amplitude than the ERP waveform, the technique of averaging highlights the ERPs and reduces the noise. This is the easiest strategy currently used to detect ERPs, also used in this paper as a reference method to be compared with our new method to calculate ERPs.

In general, to calculate ERPs by the method of averaging, essentially three conditions or hypotheses must be satisfied [27]:

1. The signal is time-locked and waveform-locked.
2. The noise is uncorrelated with the signal.
3. The latency is relatively stable (low jitter).

The epochs’ time-locking depends only on a simple technical procedure, whereas stability of the waveform, latency, and noise are intrinsic properties of the ERP. Intuitively, the averaging can capture only the ERP components that repeats consistently in latency and phase with respect to an event (the stimulus). Otherwise, the differences in phase could cause the partial cancellation of the averaged ERP.

The new method also requires these three conditions, but it is less restrictive about the stability of the phase and latency, and it is also less sensitive to residual artifacts present in the EEG signals.

The averaging of epochs is nevertheless only the last step in the calculation of the ERP.

Several pre-processing stages are usually necessary because the EEG signals are prone effects from important artifacts such as eye movements, heartbeat (ECG artifacts), head movements, bad electrode-skin contacts, line noise, fluorescent lamps, etc. All these artifacts can be several times larger (up to 10–20 times or more) than the underlying ERPs; therefore, they are able to alter calculated averages with random waves and peaks which can hide the true ERP waveform.

The first most used pre-processing step includes a band-pass filter in the range of 0.5 to 30 Hz obtained with a digital filter, which must not change the signal phase [4]. The reverse Fourier transform is suitable for this purpose, among other methods. Many researchers have suggested that the P300 component is primarily formed by transient oscillatory events in the range which includes delta, theta, and alpha, and therefore, a 1 to 20 Hz band-pass could be sufficient [11, 30].

The successive step includes a variety of methods: among the most used is the independent component analysis (ICA) algorithm [19, 28] which allows separating the true EEG signal from its undesirable components (twitches, heartbeat, etc.). In general, this method requires a decision on which signal component (after separation) is to be chosen.

One of the most common problems is the removal of ocular artifacts from the EEG signals, for which purpose several techniques were developed based on the subtraction of the averaged electro-oculograms and also on autoregressive modeling or adaptive methods [9, 10, 14].

Blind source separation [16] is a technique based on the hypothesis that the observed signals from a multichannel recording are generated by a mixture of several distinct source signals. Using this method, it is possible to isolate the original source signal by applying some kind of transformation to the set of observed signals.

Discrete wavelet transform (DWT) is another method that can be used to analyze the temporal and spectral properties of non-stationary signals [13, 21, 29]. Features in both time and frequency as well as time-frequency domain can be extracted using DWT, which has already been recognized as a very good linear technique for analysis of non-stationary signals such as EEG signals [12].

The artificial neural network, known as adaptive neuro-fuzzy inference system, was described as useful for P300 detection [23]. Moreover, the adaptive noise canceller and adaptive filter can also detect ERPs [1, 3].

A good description of the ERP technique and wave components is made by Steven J. Luck [27].

### Synchronization in EEG signals

The synchronization of neural assemblies has been widely utilized mainly in human EEG studies of brain function and disease [20, 22]. The synchronization phenomena have been increasingly recognized as a fundamental feature for the communication between different regions of the brain [7].

In this paper, the concept of EEG correlations between the EEG channels was proposed as alternative method to calculate the ERPs. Several methods were developed for quantifying relationship between time series, for example: Pearson product-moment correlation, Spearman rank-order correlation, Kendall rank-order correlation, mutual information [7], cross correlation, coherence, and wavelet correlation [20].

In our method, we use the Pearson correlation, defined as:

1 $$ r={\scriptscriptstyle \frac{\mathrm{COV}\left(A,B\right)}{\sqrt{\mathrm{var}(A)*\mathrm{v}\mathrm{a}\mathrm{r}(B)}}} $$

where *A*(*t*) and *B*(*t*) are two time serie, COV(*A*, *B*) is the sample covariance, and var(*A*) and var(*B*) are the respective sample variance. Correlation can take any value in the range (−1, 1) and in particular a value near +1 means that the two time series (i.e., two EEG channels) are strongly in phase, a value −1 means that the two signals are in opposition of phase, and a near-zero value indicates no relationship. The Pearson correlation was selected because the calculation of *r* is simple, fast, and fully independent from the absolute amplitude of the EEG signals, and then it represents only the variations of phase-correlation between two or more EEG channels.

## Materials and methods

### EEG instrument

The EEG signals were recorded using a low-cost EEG device, the Emotiv EPOC® EEG neuroheadset. This is a wireless headset and consists of 14 active electrodes and 2 reference electrodes, located and labeled according to the international 10–20 system. Channel names are AF3, F7, F3, FC5, T7, P7, O1, O2, P8, T8, FC6, F4, F8, and AF4. The acquired EEG signals are transmitted wirelessly to the computer by means of weak radio signals in the 2.4 GHz band. The Emotiv’s output sampling frequency is 128 Hz for every channel, and the signals are encoded with a 14-bit precision.

Moreover, the Emotiv hardware operates preliminarily on signals at higher sampling frequency with a digital signal processor (DSP) and performing a band-pass filtration from 0.1 to 43 Hz; consequently, the output signals are relatively free from the 50/60 Hz power-line frequency; however, they are often rich in artifacts.

The Emotiv EPOC® headset was successfully used to record ERPs [5] although it is not considered a medical-grade device. Emotiv EPOC® was moreover widely used for several researches in the field of brain-computer interface (BCI) [8, 18].

We collected and recorded the raw signals from the Emotiv EPOC® headset using software we created ourselves and saved in the .CSV format. The same software we created was used to give the necessary auditory and/or visual stimuli to the subject.

### Participants

Subjects were ten healthy volunteers, ranging in age from 28 to 69 years, informed in advance about the experimentation’s purpose. Each participant gave written consent, with Institutional Review Board (IRB) approval. Participants had normal vision and no history of hearing-related problems; they were resting in a comfortable position during the tests.

### Experimental protocol

Firstly, using a proprietary Emotiv EPOC® software, the impedance of the skin-electrode contact was kept lower than 10 kΩ in order to record better EEG signal.

The ERPs were induced by an auditory stimulus (pure 500 Hz sine wave) with a simultaneous light flash using an array of 16 red high-efficiency LEDs. The stimulus length was 1 s, and the stimuli were repeated 128 times with an inter-stimulus interval ranging randomly from 4 to 6 s.

Using the original EEG reference electrode of the Emotiv EPOC® headset (mastoidal), we recorded a first group of experimental data on 14 channels. Another group of better quality EEG files were recorded with the reference electrodes connected to the earlobes, a variation that assures better quality of the signals, rather than in the standard configuration of the Emotiv EPOC® headset where the reference electrodes are placed on an active area of the head.

## The new algorithm

In this paper, the GW6 method is described step-by-step, as well as using a procedure written in Matlab programming language (see Additional file 1: Appendix).

With our software, we pre-processed the EEG files using digital data-filtering in the 1 to 20 Hz band followed by a method we called normalization.

The filtering was performed using the reverse Fourier transform which does not change the signal phase. The conservation of the original phase of signals is very important for the application of our method. On the other hand, the conservation of the information about the phase pattern of the signals, rather than the simple power of the signals, was found important also in the representation of semantic categories of objects, especially in the low-frequency band (1 to 4 Hz) [6].

The second step in pre-processing was signal normalization: the raw signal from each channel, i.e., *S*(*x*) where *x* is the sampling index along 4 or 5 s epochs, was normalized as:

$$ S\mathit{\hbox{'}}(x)\frac{K*\left[S(x)-S\right]}{\sqrt{\left(\frac{1}{N}*{\displaystyle \sum_{x=1}^N{\left(S(x)-\overline{S}\right)}^2}\right)}} $$

where *S* is the mean of *S*(*x*) in the epoch.

The signal *z*-score is then multiplied by a factor *K*, where *K* is an experimental constant which restores the averaged optimized amplitude of the EEG signal. The *K* factor is the standard deviation of a good quality EEG signal, found experimentally using this specific instrument. This number was calculated as *K* = 20, and this normalization step created an epoch with a shape identical to that of the original EEG signal, but transferred into a uniform scale, with comparable amplitude for every epoch. Moreover, this normalization step do not changes the phase correlation among all the EEG channels. The entire file fully processed as such was saved as new file in .CSV format, containing all the information about the start and the end of each stimulus.

Note that it is also possible to pre-process only time-locked epochs, for example, 3 s long, corresponding to each stimulus [pre-stimulus + stimulus (1 s) + post-stimulus], and in general, this procedure gives non-identical results although very similar to the previous method based on the filtering and saving of the entire file.

Another common way to pre-process the data for the ERP calculation is the exclusion of every epoch with an amplitude above a fixed threshold, for example, 80 μV. A disadvantage of this technique is that a large number of epochs could be discarded and consequently the average could be calculated on insufficient data. In our software, we also used this procedure to eliminate epochs with strong artifacts above 100 μV still present after the digital filtration.

In this paper, we will illustrate a new method which is useful for detecting ERPs even among particularly noisy signals and with significant latency variations, known as “latency jitter”.

Our method, called GW6, is less restrictive regarding the issue of jitter, and allows ERP detection when the standard approach, based on the average, fails or gives unsatisfactory results due to several artifacts. However, the new method does not reproduce the typical biphasic waveform of the ERP but rather an always positive waveform. For this reason, this new procedure is useful if used together with the classic technique of averaging, rather as an alternative to the latter.

The new method uses Pearson’s correlation extensively for all EEG signals recorded by a multichannel EEG device. By using the method of averaging, it is possible to work with a single EEG channel too, whereas the GW6 method works only with a multichannel EEG device, starting from a minimum of about six channels. Nevertheless, it is also possible to calculate the ERP for each channel as in the standard method.

In many papers describing a mathematical method to analyze something, formulas are usually given, which must be subsequently translated into a computer-language, for example C, C++, Visual Basic, Java, Python, Matlab, or other. This step could be difficult and limit the release and application of some useful methods. In this paper, we will describe this new algorithm as a step-by-step procedure and also in the simple and well-known Matlab programming language, in order to ease its application (see Additional file 1: Appendix).

**Fig. 1:**
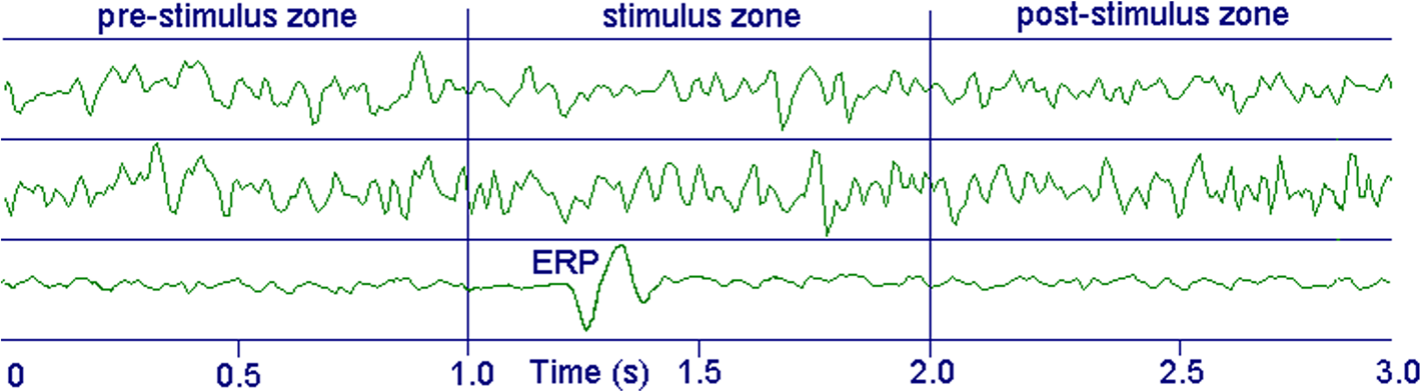
The two *upper tracks* represent the raw signals of two EEG channels in time-locked epochs, whereas the *lower track* is the average of a sufficient number (about 100) of epochs for each channel (ERP is not to scale). The figure shows a positive peak about 300 ms after the stimulus’s onset (P300 wave). The ERP’s typical duration is about 300–500 ms, depending on the kind of stimulus and band-pass filtering of the signal

**Fig. 2:**
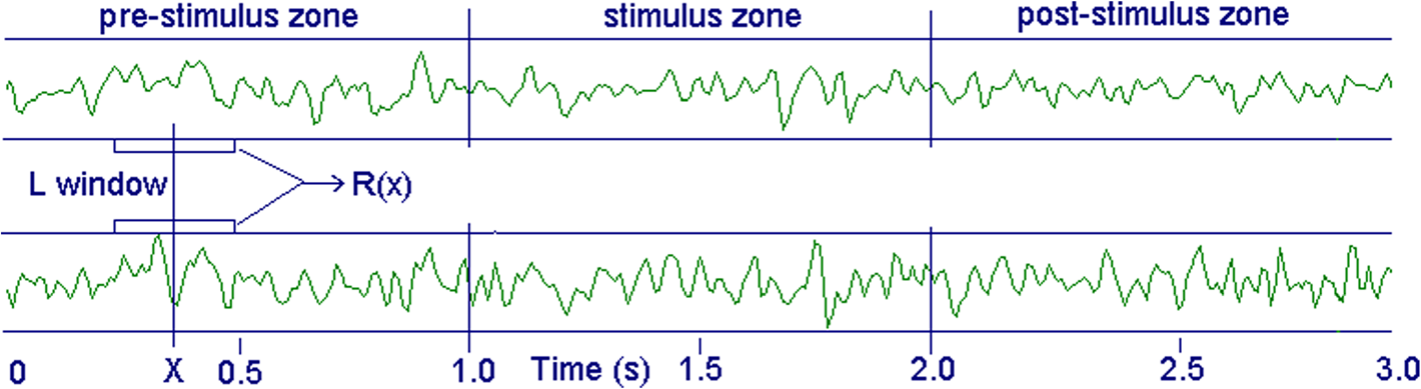
The double data-window lasting L is shifted progressively along the two tracks S1(*x*) and S2(*x*), and the corresponding Pearson’s correlation between the two windows is calculated and stored in the vector *R*(*x*), where *x* is the sampling data index of the tracks

We describe the basic idea of this new method in Figs. 1 and 2.

Let us now consider the Fig. 2 and the double data-window lasting L (about 270 ms, 34 samples) centered at point *X* of the signal. We can calculate the linear Pearson’s correlation between these two data segments, and the result will be a number *r* represented by the vector *R*(*x*), which can be calculated for every point *X* simply by progressively moving the windows along by one sample unit at a time (sliding windows). In general, the averaged value of *R*(*x*) will vary from the pre-stimulus zone to the stimulus zone because the (auditory or/and visual) stimulus changes the correlation between the two EEG signals, which represent the activity of different parts of the brain. An interval about 270 ms long was selected because it represents the typical amount of time required for a conscious response corresponding to the P300 wave, but different intervals could be selected for fast Evoked potentials, or other types of stimulus.

This change of correlation can appear either as an increase or a decrease with respect to the baseline (i.e., the zone preceding the stimulus). Let us consider a real example, based on the Emotiv EPOC®, where the number of channels is NC = 14, the sampling frequency is 128 samples/s, the stimulus length is 1 s, and the epoch length is 3 s. In this case, it is possible to calculate the vector *R*(*x*) in a number of pair combinations Nt = NC_*_(NC − 1)/2 = 91 for each stimulus (epoch).

The result could be expressed using a new array *R*(*I*, *X*) where *I* = 1… 91, and *X* = 1… 384.

This last number arises from a 3-s length epoch and 128 samples/s, with the stimulus given at sample number 128, and stopped at sample number 256, after an extra second.

Each value of *R*(*I*, *X*) comes from the Pearson correlation between two data-windows of duration L (i.e., 34 samples) centered on point *X*, and for any pair combination of the NC channels.

Moreover, the array *R*(*I*, *X*) is averaged along all the Ns stimuli given to the subjects.

In general, we can represent the raw signals as a time-locked array of *V*(*C*, *X*, *J*) type, where *C* = 1… 14 are the EEG channels, *X* = 1… 384 are the samples along 3 s, and *J* = 1… Ns is the number of stimuli given to the subject, usually about 100 or more. The entire GW6 procedure is better described in the Matlab method (see Additional file 1: Appendix).

The following are the processing stages based on the 14-channel Emotiv EPOC® device, but not limited to this specific device (the numbers here described are only examples):

Stage 1: filtration of the .CSV file in the frequency band 1–20 Hz, normalization, and new saving of the entire file. It is however possible to omit this stage and go directly to filtration and normalization on the time-locked epoch of each stimulus of the file.

Stage 2: from the raw EEG data, or from the pre-processed file, calculate all the time-locked epochs and storage of the data in the array *V*(*C*, *X*, *J*), where *C* = 1… 14 are the channels, *X* = 1… 384 are the samples, and *J* = 1… Ns is the stimulus index. However, due to the presence of the L windows, we need a longer array for processing, for example, the length could be increased by two tails of length *L* = 34, giving a total number of *L* + 384 + *L* = 452 samples, with the stimulus starting at *X* = 162 and stopping at *X* = 290. Now the array *V*(*C*, *X*, *J*), filtered and normalized, is renamed as the new array *W*(*C*, *X*, *J*).

$$ V\left(C,\ X,\ J\right)+\mathrm{filtration}+\mathrm{normalization}\to W\left(C,\ X,\ J\right). $$

It is very important that any pre-processing method modifying the correlation among the signals must be excluded.

Stage 3: calculation of the simple average of *W*(*C*, *X*, *J*) among all Ns epochs (number of stimuli), giving the final array Ev(*C*, *X*), which is the simple and classic ERP of each channel.

$$ \mathrm{E}\mathrm{v}\left(C,X\right)=\frac{1}{\mathrm{Ns}}*{\displaystyle \sum_{J=1}^{J=\mathrm{N}\mathrm{s}}W\left(C,X,J\right)} $$

A detail to note: when processing has finished, the *X* index is easily recalculated in order to cut off the tail lengths *L* at the beginning and end, giving the final array Ev(*C*, *X*) where *X* = 1…384 and *C* = 1…14.

This array Ev(*C*, *X*) is used in this paper as a comparison with the result of our method and to show the differences in the waveform of the resulting ERP.

Stage 4: calculation of all the Pearson’s correlation combinations using a sliding-window 270 ms long, as described in Fig. 2. The result is the array *R*(*I*, *X*), where *I* is the index of pair combinations, which is finally calculated as the average of all the stimuli. Here too, at the end of this stage, the index *X* is recalculated in order to cut off the initial and final *L* tails, giving the final array *R*(*I*, *X*) where *I* = 1… 91 and *X* = 1… 384 (see Additional file 1: Appendix). This array is the average from all the Ns stimuli.

According to Eq. 1, we can describe this stage also using this formal expression:

$$ R\left(I,X\right)=\frac{1}{\mathrm{Ns}}*{\displaystyle \sum_{J=1}^{j=\mathrm{N}\mathrm{s}}\mathrm{Pearson}\left(I,X\right)} $$

where Pearson(*X*, *I*) is the *r* Pearson value calculated from *X* = 1 to N (1..384); t from (X-L/2) to (X + L/2) is the time series, and *A*(*t*) *W*(Ca, *X*, *J*); *B*(*t*) *W*(Cb, *X*, *J*); *I* any pair combination Ca and Cb of the C channels.

Stage 5: Calculation of the baseline Bs(*I*) mean value for each combination, described by the *I* index of the array *R*(*I*, *X*); *I* = 1…91 (Nt = 91). The best baseline is calculated as a balanced average of pre-stimulus plus post-stimulus of each combination:

$$ \mathrm{B}\mathrm{s}(I)=\frac{1}{\left(N+b1-b2\right)}*\left({\displaystyle \sum_{x=1}^{b1}R\left(I,X\right)+{\displaystyle \sum_{x=b2}^NR\left(I,X\right)}}\right) $$

where b1 is the stimulus start temporal index, and b2 is the stimulus end, then subtracting this baseline from the array *R*(*I*, *X*), and taking the absolute value:

$$ R\mathit{\hbox{'}}\left(I,X\right)=\left|\left(R\left(I,X\right)-\mathrm{B}\mathrm{s}(I)\right)\right| $$

The absolute value is calculated because it allows the simple average among all the Nt combinations (see Stage 6). In fact, the variation of correlation during the stimulus can give both positive or negative changes of *R*(*I*, *X*) for each *I*, and only taking the absolute value the average (Stage 6) is always positive.

**Stage 6**: average along all the Nt combinations (and all the stimuli), giving the final array

Sync1(X):

$$ Sync1(X)=\frac{1}{Nt}*{\displaystyle \sum_{I=1}^{I=Nt}R\mathit{\hbox{'}}\left(I,X\right)} $$

which represents the global variation of the EEG correlations during a 3-s epoch.

For the reason described at Stage 5, this variation appears always as positive peak.

It is also possible to calculate an equivalent array Sync2(*C*, *X*) for each channel *C* (see Additional file 1: Appendix).

$$ \mathrm{Sync}2\left(C,X\right)=\frac{1}{\left(NC-1\right)}*{\displaystyle \sum_{I=\left(C,K\right)}R\mathit{\hbox{'}}\left(I,X\right)} $$

where *I* is the index of all the channel *C* pair combinations with any other *K* channel. The number of these combinations is (NC-1) for every channel.

**Fig. 3:**
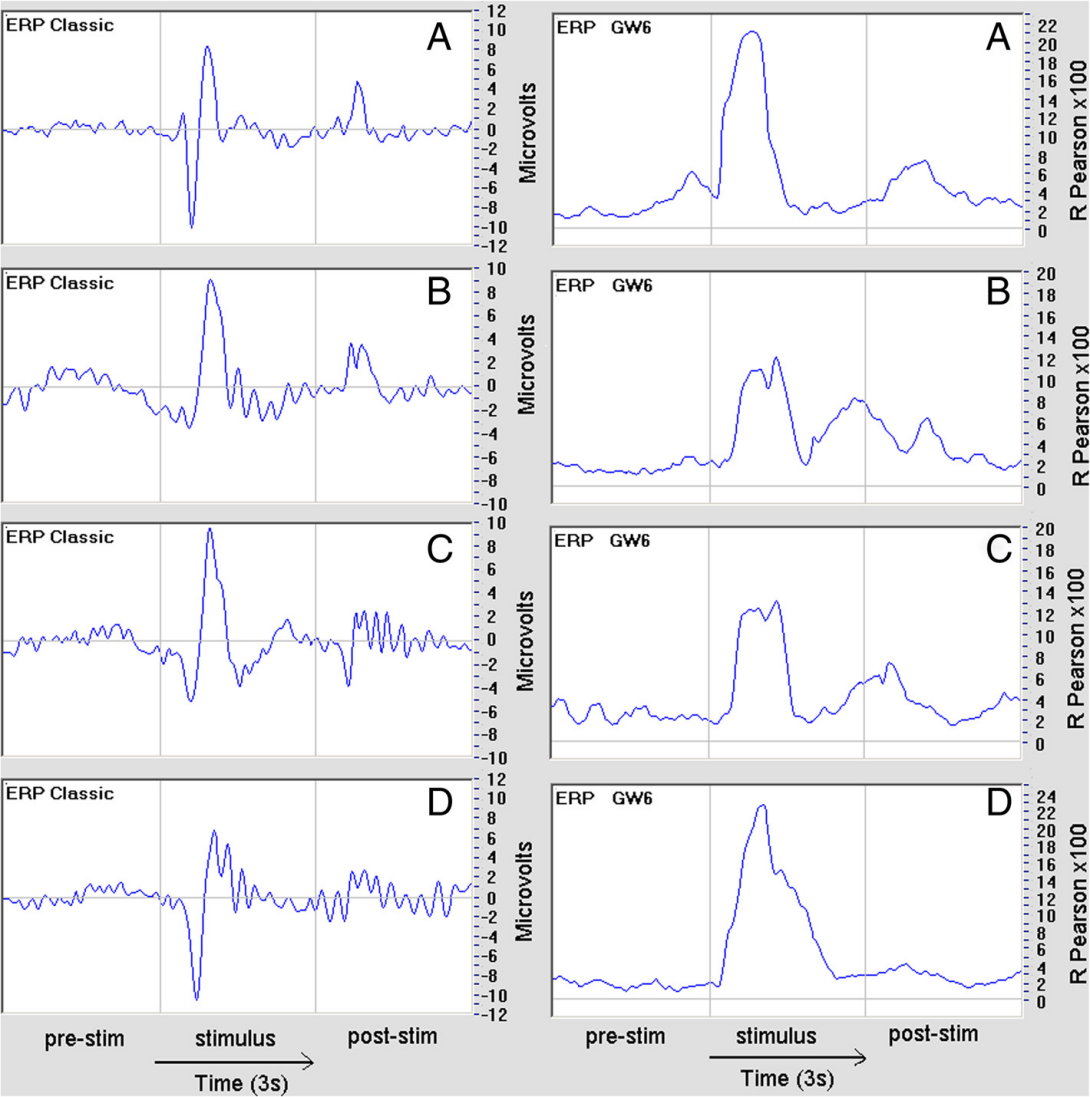
In these pictures, shown as examples, the *left* presents the classic ERP (amplitude in microvolts). On the *right* is shown the corresponding GW6 graph; the result is expressed as the R-Pearson value multiplied by 100. All these graphics are the global average of 14 EEG channels and about 120 stimuli; the EEG data were filtered in the 1–20 Hz band and submitted to the normalization routine. In all cases, a positive peak is observed coinciding with the P300 maximum peak, but in the majority of cases, the positive peak of the GW6 graph is larger than the corresponding classic ERP (see, for example, cases B, C, and D)

The global array Sync1(*X*) and Sync2(*C*, *X*) will show one or more positive peaks in the ERP zone, as shown in Fig. 3, and these peaks represent the variations of correlation among the different brain zones (electrodes) during the stimulus.

## Experimental results

**Fig. 4:**
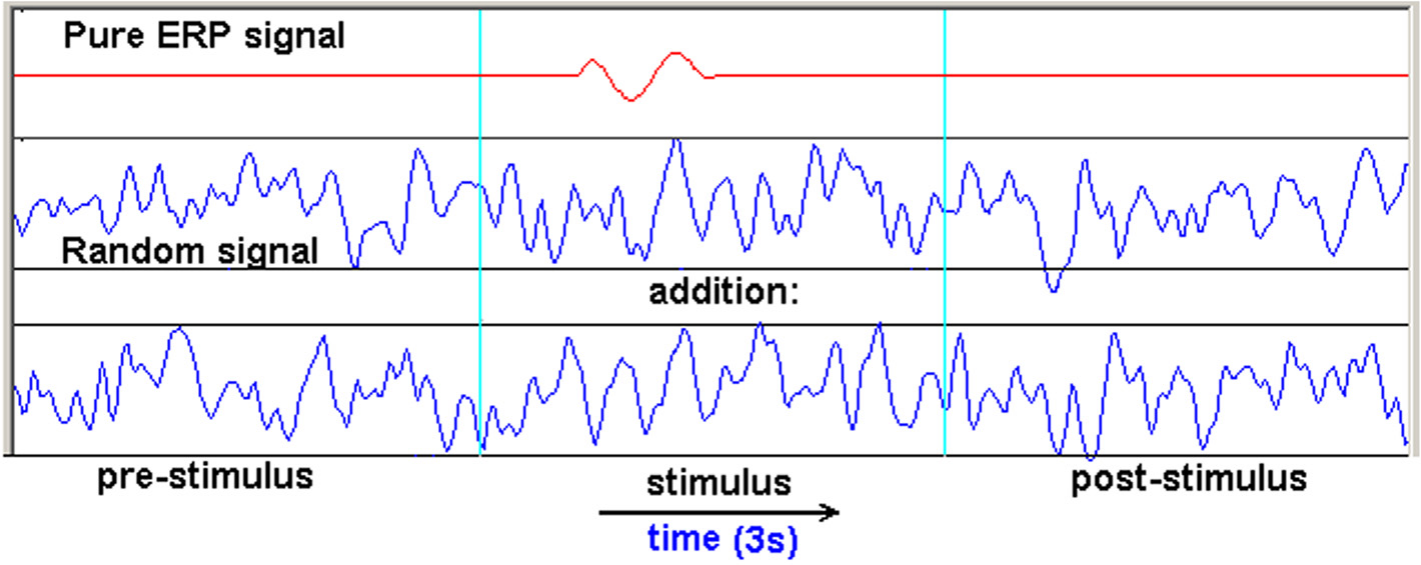
Artificial ERP signal mixed with a variable amount of a random signal and submitted both to the classic average and to the GW6 routine

These graphs are examples of the typical results provided by this method:

In order to better investigate the properties of the GW6 method, we wrote an emulation software. In this software, a simple artificial ERP waveform was added to a random noise and suitably filtered (low-pass filter) in order to reproduce the typical frequency distribution of the EEG signal. The artificial ERP signal was mixed with a variable amount of this random signal, and the result was submitted both to the classic average and to the GW6 routine (Fig. 4).

**Fig. 5:**
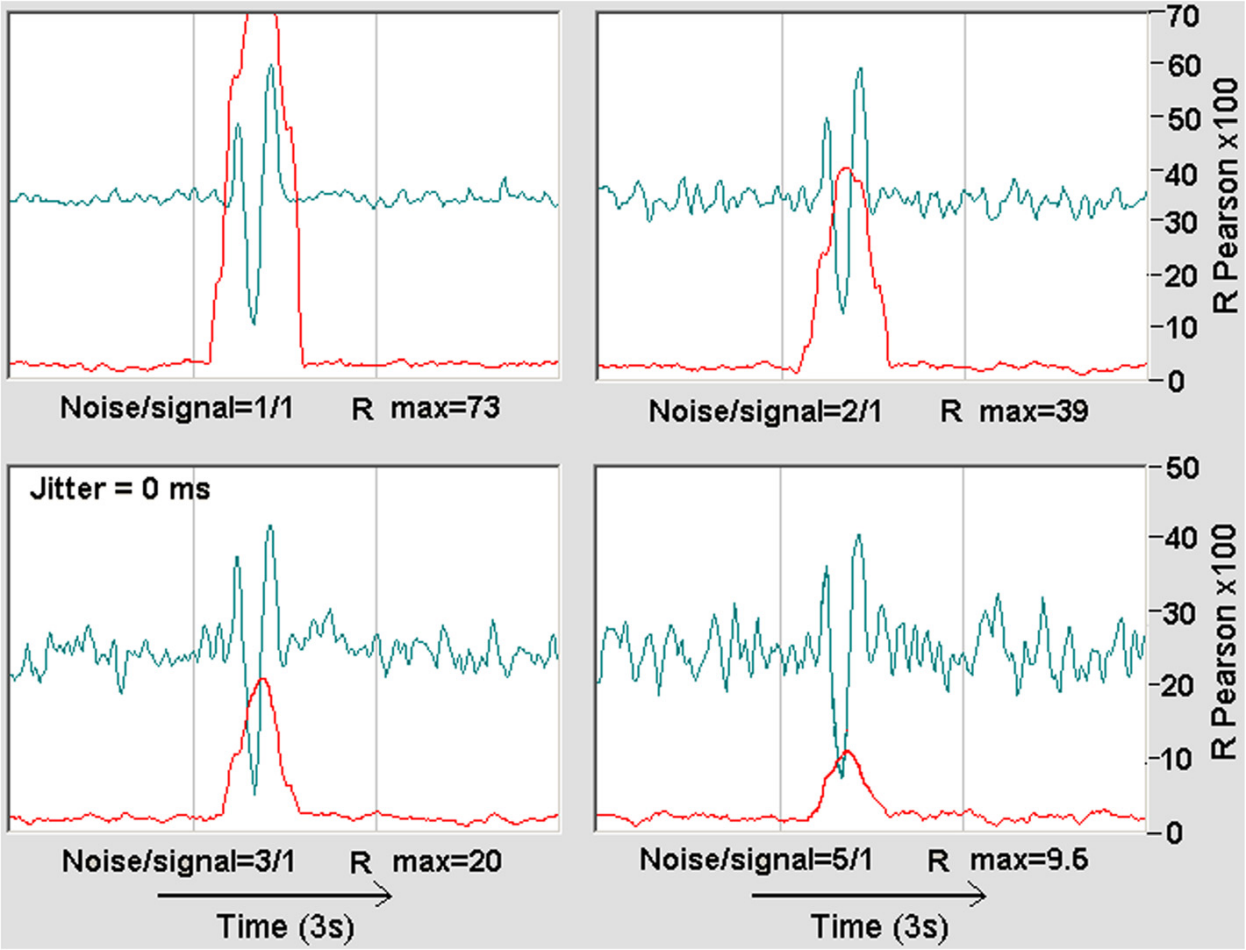
Example of the ERP + random signal emulation for four levels of noise-to-signal ratio

Figure 5 shows the results of the classic average method and of the GW6 method for a progressive increase of the noise-to-signal ratio, as an average of 100 ERPs on a single channel. While the final amplitude of the ERP waveform does not change, but instead becomes progressively noisier, the GW6 graph’s amplitude (red curve) progressively drops, but with a stable residual noise.

Moreover, the width of the red curve is approximately equal to the width of the classic ERP (all peaks included). Not only the waveform of GW6 graph change little using a L windows of about 150 ms rather than 270 ms as described in the previous section. In general, the larger the amplitude from classic ERP is, the larger correlation would be observed using our new method. But the relation is not linear and is depending from the noise of the EEG signal.

Of particular interest is the emulation of these two methods in the presence of the so-called latency jitter, which is an unstable ERP time latency that in some cases could affect the ERPs.

**Fig. 6:**
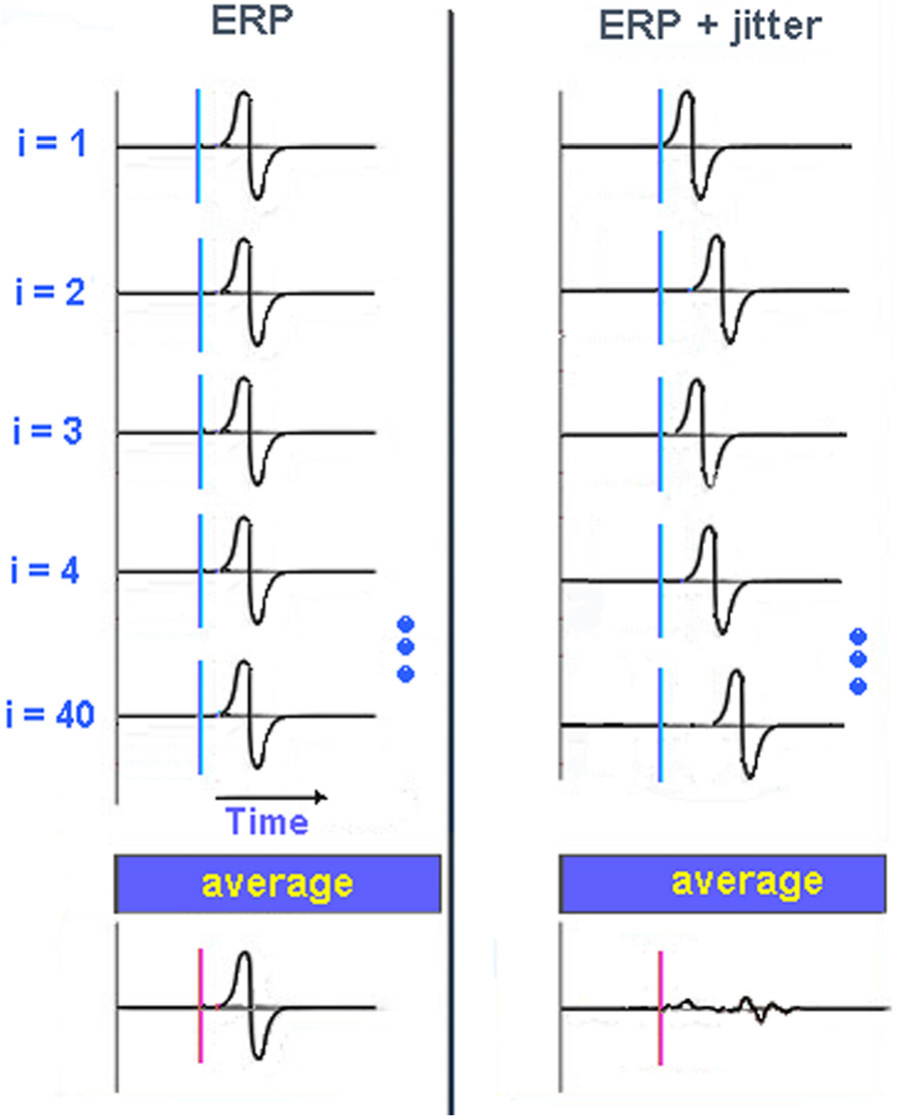
ERP with stable latency on the *left* and with latency jitter on the *right*

**Fig. 7:**
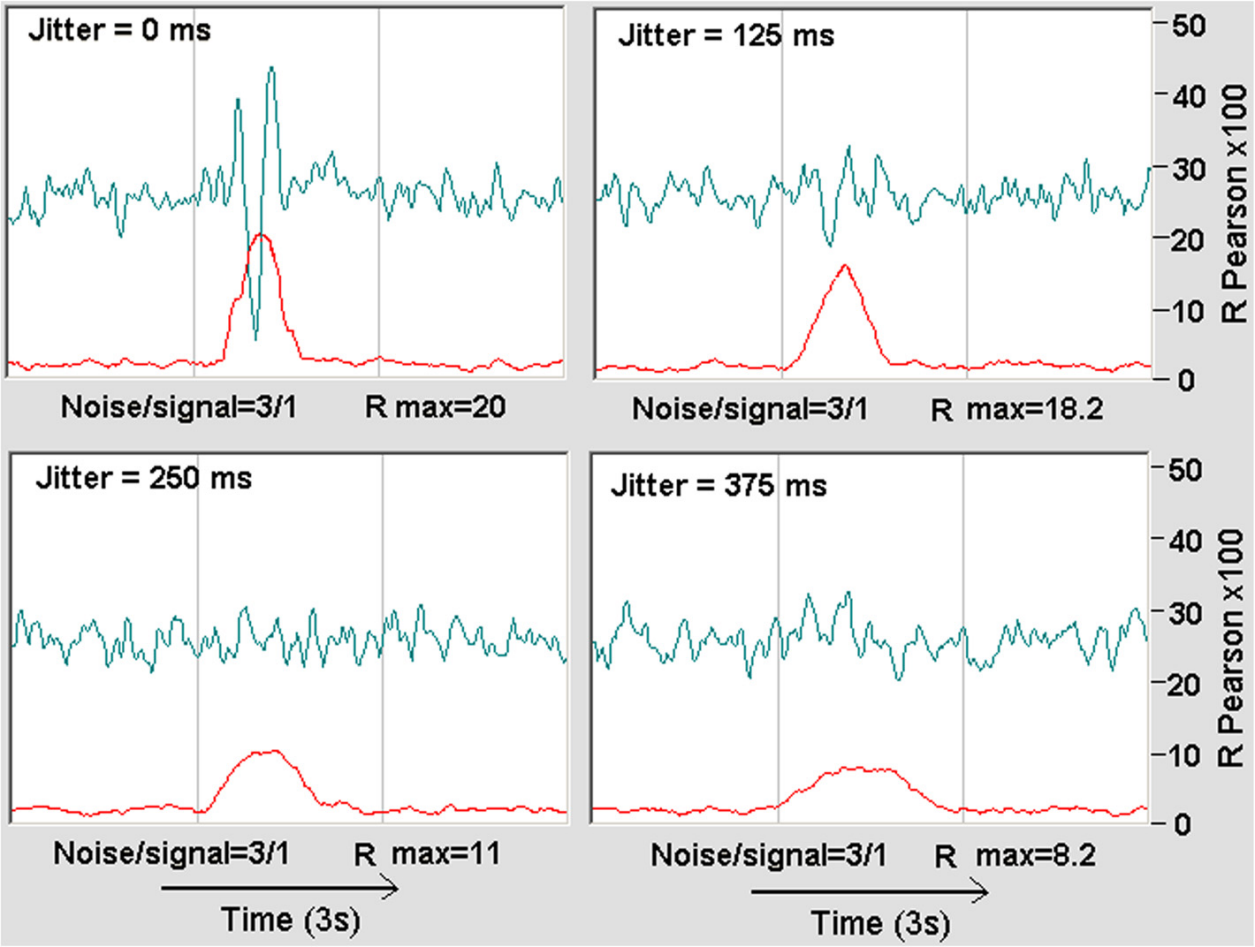
A stable noise-to-signal ratio (3/1), but with a random jitter progressively increased

When the ERP latency is stable (Fig. 6, left picture), its average is stable too and is at its maximum amplitude. Nevertheless, if latency jitter is present (due to some physiological cause), the corresponding average decreases because each ERP does not combine with the same phase and consequently ERPs have a tendency to cancel each other out. This effect is more pronounced as the jitter increases. In the software emulation of Fig. 7, a stable noise-to-signal ratio (3/1) was used, but with a random jitter progressively increased. Moreover, the jitter was random between the ERPs, but was constant for all the channels in each ERP. The results show that the GW6 routine is more resistant to jitter than the simple classic average.

Whereas the classic ERP waveform disappears rapidly as the jitter increases, the GW6 routine gives a still identifiable result (the red curve), where the amplitude is decreases but not as rapidly, and the curve’s width is increases. This interesting property is very important, because it suggests some other possibility about the large GW6 peaks observed in Fig. 3, in particular in B, C, and D cases.

**Fig. 8:**
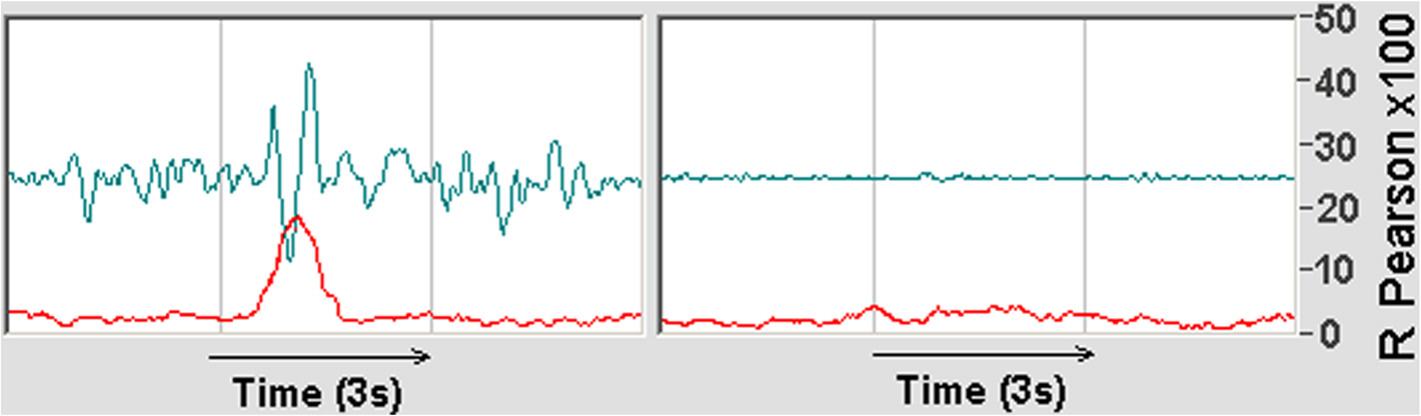
Case 1. *Left*: *W*(*C*, *X*, *J*) from ERP pure wave + random noise, Jitter = 0, average of 100 ERPs. *Right*: with the same processing of the corresponding *W*'(*C*, *X*, *J*) array both graphs disappear

**Fig. 9:**
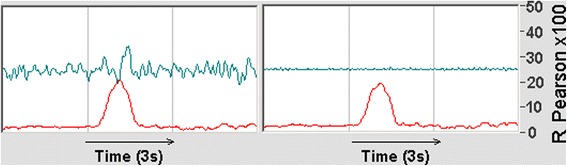
Case 2. *Left*: *W*(*C*, *X*, *J*) from ERP pure wave + random noise, Jitter = 78 ms (from 0 to 78 ms, random), average of 100 ERPs. *Right*: with the same processing of the corresponding *W*'(*C*, *X*, *J*) array only the classic ERP disappears. In the presence of Jitter, the GW6 method always shows an ERP

Following a hunch, we added a new and simple process to our software used to analyze the true ERP using both the classic and the GW6 methods. At the end of the process, which gives the typical result shown in Fig. 3, we created another procedure where the classic ERP average was subtracted (see Additional file 1: Appendix) from the set of EEG signals *W*(*C*, *X*, *J*), giving a new array:

*W*'(*C*, *X*, *J*) = *W*(*C*, *X*, *J*) − Ev(*C*, *X*), then this new data-set was submitted to Stages 3, 4, 5, and 6 previously described. Incorporating this process in our emulation software, and successively performing the same 3, 4, 5, and 6 stages, no ERP appears as a result nor does any significant GW6 peak. This is obvious because in doing so we have canceled the ERP component from the random noise, and consequently, nothing is expected to appear, but that is true only if jitter is zero (Figs. 8 and 9).

**Fig. 10:**
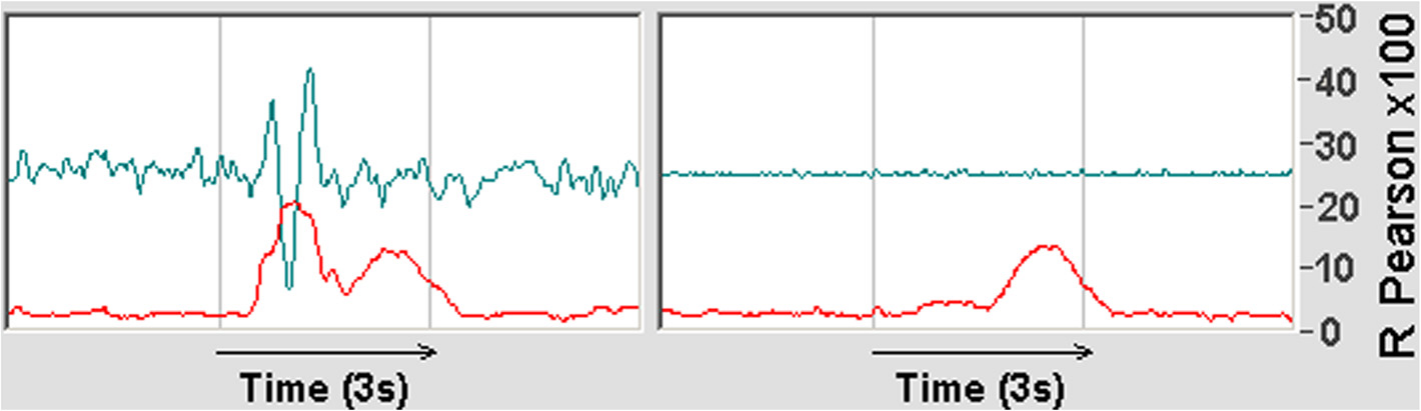
Case 3. *Left*: *W*(*C*, *X*, *J*) from ERP pure wave + random noise, Jitter = 0, RCS width about 400 ms, average of 100 ERPs. *Right*: with the same processing of the *W*'(*C*, *X*, *J*) array only the classic ERP disappears, not that due to RCS

We created a new variant in the emulation software: alongside the pure ERP wave + random signal, we also added a random common signal (RCS) to every channel only in a limited zone near the ERP, but this RCS is random between the ERPs (Fig. 10)*.* In this emulation variant, we hypothesized that the stimulus given to the subject could not only cause a simple brain response based on a stable waveform with low jitter (the classic ERP) but also cause a non-stable waveform very similar or identical in all the EEG channels at each stimulus*.* A simple calculation of the average does not reveal this kind of electrical response, because the waveform is near random, but it is easily revealed by the GW6 method, which is based on the calculation of the variation of correlation among all the EEG channels during the stimulus.

**Fig. 11:**
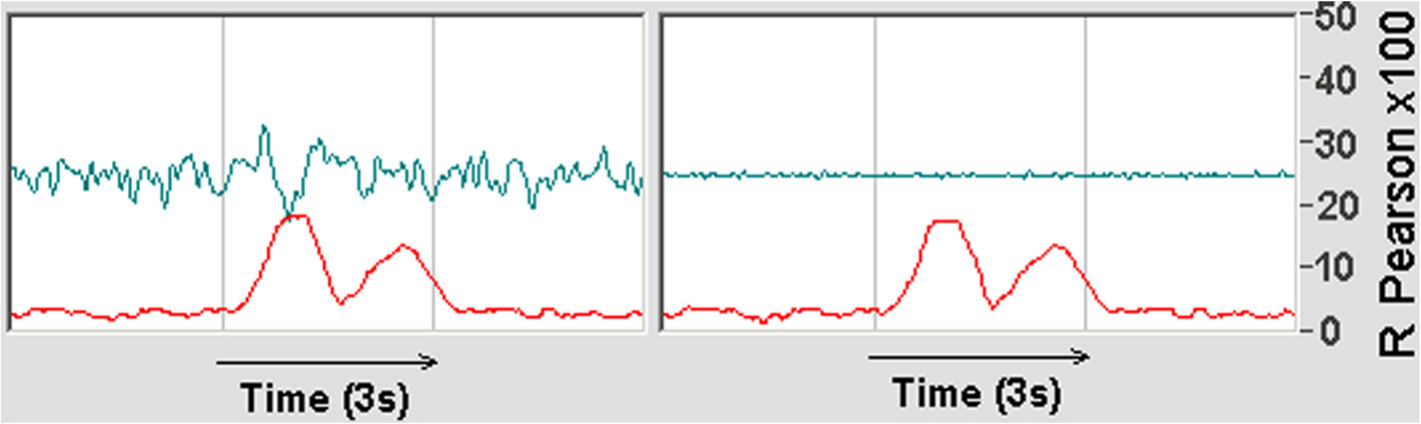
Case 4. *Left*: *W*(*C*, *X*, *J*) from ERP pure wave + random noise, Jitter = 78 ms, RCS width about 400 ms, average of 100 ERPs. *Right*: with the same processing of the *W*'(*C*, *X*, *J*) array, now both peaks are visible

**Fig. 12:**
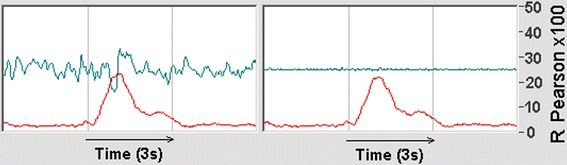
Case 5. *Left*: *W*(*C*, *X*, *J*) from ERP pure wave + random noise, Jitter = 78 ms, RCS width about 860 ms, average of 100 ERPs. *Right*: with the same processing of the *W*'(*C*, *X*, *J*) array, now both the peaks overlap and are visible

**Fig. 13:**
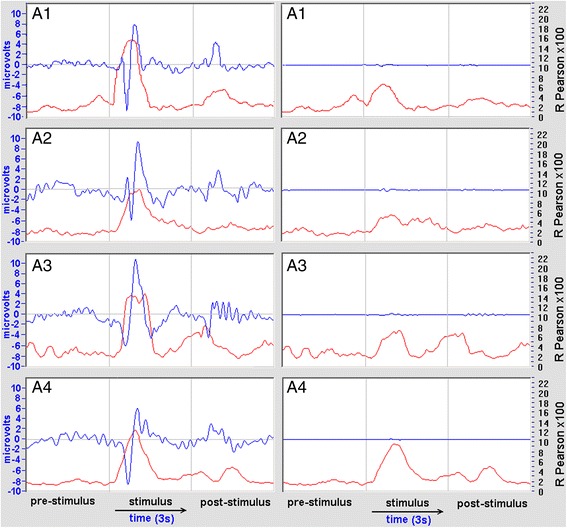
Results of the true experimental ERP analysis of the *W*(*C*, *X*, *J*) data followed by transformation into the *W*'(*C*, *X*, *J*) data-set and new method GW6

We believe that the two last cases (4 in Fig. 11 and 5 in Fig. 12) best represent true experimental ERPs. With our emulation software, many combinations and situations can be calculated. Now, if we submit our true experimental ERPs to the same procedure, i.e., analysis of the *W*(*C*, *X*, *J*) data followed by transformation into the *W*'(*C*, *X*, *J*) data-set and a new analysis, we obtain these typical results (Fig. 13).

As shown in Fig. 13, in the majority of cases, after the subtraction of the classic ERP waveform from the EEG data, the GW6 method (red graph) shows a reduction in amplitude corresponding to the standard ERP wave, but other peaks are hardly changed at all, and in several cases, there is minimal change to the whole graph.

## The ERP decomposition in sub-bands

In a recent paper, Ahirwal et al. [2] proposed to decompose the ERP signal into the conventional bands delta, theta, alpha, and beta in order to extract feature corresponding to each band and to calculate the Combined Factorised Feature Extraction (CFFE).

The purpose is to increase the control commands for applications in the important area of brain-computer interface (BCI).

The new method here described works also very well when it is applied to an ERP signal pre-filtered in any sub-band. Very important, the filtering must be performed using any kind of digital filter that does not change the signal phase.

**Fig. 14:**
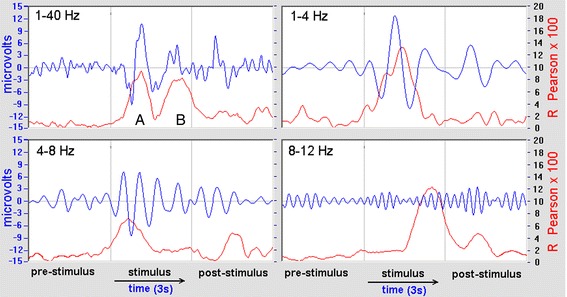
When the new method is applied to EEG sub-band, some peaks (like A and B) in the full-band 1–40 Hz can be splitted like in this example, with the peak A being mainly represented in the band 1–8 Hz, and the peak B mainly in the alpha band. The standard ERP on the contrary does not show any peak in the alpha band

In order to test the behavior of our method in this case too, we filtered the EEG signals in the full-band (1–40 Hz), then in delta (1–4 Hz), theta (4–8 Hz), and alpha (8–12 Hz). Then we calculated the ERP by GW6 and compared the result with the standard averaging procedure. Surprisingly, we observed that several subjects endowed with an intrinsic medium-high level of alpha rhythm show the tendency to generate an ERP (in the full-band) with two main peaks, the first at about 300 ms from the stimulus onset, the second at about 600–800 ms (see Fig. 14).

The subjects with low alpha rhythm (determined by the simple averaged FFT of the normalized EEG, as previously described) in general show only the dominant peak at about 300 ms.

The new method seems able to identify correlations (peaks) in bands and with latency not easy identified by the simple standard averaging. Several questions arise from this observation: why the latency of alpha ERP is so different from about 300 ms? Why it is observed mainly in subjects with high spontaneous alpha rhythm? But the purpose of this paper is not, at present time, to inquire about these questions.

## Discussion

This new method allows the calculation of ERPs as variations of the global correlations among all the EEG channels, with respect to the averaged pre-stimulus and post-stimulus zone.

The basic idea is a sliding-window of Pearson’s correlation between two EEG channels in the ERP zone, in any pair combination.

The method should not be regarded as alternative to the classic averaging calculation but as an integration and expansion of the information that we can draw out from the EEG data.

Furthermore, the method shows significant peaks in the P300 zone larger than the peaks calculated from the standard procedure of averaging. In the presence of significant jitter (instability of latency), the new method is superior with respect to the classic one and shows significant peaks in this case too. Our experimental results suggest that, in the majority of cases, there is some amount of jitter coinciding with the classic ERP and/or the significant presence of other signal components that are not phase-locked, such as those hypothesized in the emulation software. These components could be easily calculated simply by subtracting the classic ERP from the EEG signal of each channel and re-analyzing the new data using the GW6 method or by filtering the EEG signal in several sub-bands.

According to Roach and Mathalon [24], we suppose that an inter-neuronal synchronization occurs on each stimulus trial, but the latency with respect to stimulus is variable across trails.

In general, it is easy to explain because the great majority of ERP components are in the lower band frequency (0.5–8 Hz). In fact, if we suppose a jitter of about 50–100 ms among trials, then this random delay is sufficient to destroy any average of frequency components near or higher than 8–10 Hz, but not in the lower frequencies, being Period = 1/Frequency. The new method seems to be significantly less sensitive to random jitter, and consequently, we can observe components (peaks) also in the 8–12 frequency range too.

Consequently, it is now possible to obtain three types of ERP: first, the classic ERP based only on simple averaging, which highlights both phase and time-locked components with low jitter; second, the new ERP which shows more components including those that are non-phase locked among trials but sufficiently in-phase among EEG channels at every trial; and third, it is possible to show only the non-phase-locked components of the ERP.

Our method is also inherently more resistant to artifacts because the Pearson’s correlation depends only on signal phase and not on amplitude, while the artifacts are mainly due to strong signal amplitude variations. Although this method is not compatible with all the pre-processing methods which change the correlation among EEG signals, it is applicable to the majority of cases, and probably also in cases not presented or discussed here due to limitations in our instrumentation and experimental setup.

## Conclusions

The purpose of this paper is not, at this time, to accurately investigate the EEG response to a specific stimulus or specific experimental protocol, but only to propose a new method for ERP detection and analysis that could become very important for future research about the nature, origin, and characteristics of ERPs in light of the preliminary result presented here.

In particular, this new method could be very useful for investigating hidden components of the ERP response, with a possible important application for medical purposes and in the fields of neurophysiology and psychology.

Furthermore, we emphasize choosing the well-known Matlab language tool for mathematical processing so that the method can be easily used with and applied to independent software as well as research.

## Additional file

Additional file 1: Appendix. (DOC 35 kb)
