# Supplementary material for: A new method to detect event-related potentials based on Pearson’s correlation
Source: EURASIP J Bioinform Syst Biol. 2016 Jun 7;2016:11. doi: 10.1186/s13637-016-0043-z (PMC4894923; doi:10.1186/s13637-016-0043-z)
Supplement: Additional file 1: — Appendix. (DOC 35 kb) [file 13637_2016_43_MOESM1_ESM.doc]

**APPENDIX**

Now suppose that the W(C, X, J) array represents all the data to be analyzed, where C are the channels (14 in the Emotiv Epoc), X the samples from X = 1 to 452, J = 1 to Ns, where Ns is the number of stimuli given to the subject (usually Ns = 100-130).

We must moreover suppose that the sampling frequency is 128Hz and we take in consideration a time-locked epoch of 3 second (pre-stimulus, stimulus, post-stimulus, 384 samples) plus two additional tails (the L windows, 34 samples) giving a total of 452 samples. These tails are necessary for the elaboration, but they will be cut off at the end, in order to give a final epoch of 3 seconds.

Moreover, we suppose that the stimulus starts at X = 162 and stops at X = 290.

Data in the W(C, X, J) array must be with zero-average (easy to implement, here omitted).

The unit of the W(C, X, J) data could be in microvolts, or raw data at 10, 12, 16 bit, etc.

% The essential core of GW6.m routine in Matlab language

V1 = zeros(1,800) ;

V2 = zeros(1,800) ;

A1 = zeros(1,800) ;

A2 = zeros(1,200) ;

F1 = 0; F2 = 0; F3 = 0; I = 0; J =0;

XM = 0; YM = 0; Rc = 0; X = 0;

Ax = 0; Bx = 0; Np = 0; Tc = 0; C =0;

Nz = 0; A = 0; U =0; X1 = 0; X2 = 0;

% definition of the value of data

N1 = 452 ; % 384 samples + 2 tails of 34 samples

NC = 14 ;

Ns = 100 ;

Np = 34 ; % 34 is a L window of about 270 ms, at 128 samples/s, could be changed

Tc = Np/2; % half window L

Nt = (NC ^ 2 - NC) / 2 ; % in our case Nt=91 is the number of combination with 14 channels

R = zeros(Nt+1,N1+1); % put zero each element of array R(I, X)

for J = 1 : Ns % for all the stimuli given

U = 0 ; I = 0;

for Ax = 1 : (NC - 1)

for Bx = (Ax + 1) : NC

I = I + 1 ; % counter of the progressive combinations of two channels

for U = Tc : (N1-Tc)

X1 = U - Tc + 1 ;

X2 = U + Tc + 1 ;

A = 0;

for X = X1 : X2

A = A + 1;

V1(A) = W(Ax, X, J) ;

V2(A) = W(Bx, X, J) ;

end

Nz = A;

correlas ; % subroutine of Pearson's Correlation

A1(U) = Rc ;

A = 0;

end

for X = Tc : (N1-Tc)

R(I, X) = R(I, X) + A1(X) ;

end

end

end

end

% averaging along all the Ns stimuli

for I = 1 : Nt

for X = 1 : N1

R(I, X) = R(I, X) /Ns ;

end

end

% cutting of the two L tails in order to recover the interval of 3 seconds

for I = 1 : Nt

U = 0 ;

for X = (Np + 1) : (N1 - Np)

U = U + 1 ;

R(I, U) = R(I, X) ;

end

end

N1 = U; % now N1 value is 384, equivalent to 3 seconds

% now the array R(I, X) is the output of this stage of elaboration

%Correlas: %%‘ Pearson’s correlation subroutine

F1 = 0 ; F2 = 0 ; F3 = 0 ; XM = 0 ; YM = 0;

for X = 1 : Nz ;

XM = XM + V1(X) ;

YM = YM + V2(X) ;

end

XM = XM / Nz ;

YM = YM / Nz ;

for X = 1 : Nz

F1 = F1 + (V1(X) - XM) * (V2(X) - YM);

F2 = F2 + (V1(X) - XM) ^ 2 ;

F3 = F3 + (V2(X) - YM) ^ 2;

end

F1 = F1 / Nz ; F2 = F2 / Nz ; F3 = F3 / Nz;

if (F2 == 0 || F3 == 0 )

Rc = 0 ;

return

end

Rc = 100 * F1 / sqrt(F2 * F3) ; % the r of Pearson is multiplied by 100

return

% successive FinalElab.m elaboration

Bs = zeros(100);

B1 = 0; B2 = 0; C = 0; J = 0; X = 0; I = 0;

% Nt = 91 is defined in the previous elaboration

B1= 128 ; B2 = B1 + 128 ; N1 = 384;

% the stimulus zone is between X = B1 and X = B2

% for the calculation of a balanced baseline, we take the pre-stimulus zone

% (from X = 1 to B1), the second is the post-stimulus zone (from X = B2 to N1)

Sync1=zeros(N1);

Sync2=zeros(NC, N1);

A=0;

for I = 1 : Nt % baseline calculation

A = 0 ; Bs(I) = 0 ;

for X = 1 : B1

A = A + 1 ;

Bs(I) = Bs(I) + R(I, X) ;

end

for X = B2 : N1

A = A + 1 ;

Bs(I) = Bs(I) + R(I, X) ;

end

Bs(I) = Bs(I) / A; % baseline for each combination

end

for I = 1 : Nt

for X = 1 : N1 ;

Sync1(X) = Sync1(X) + abs(R(I, X) - Bs(I)) ;

end

end

for X = 1 : N1

Sync1(X) = Sync1(X) / Nt ;

end

% Now the array Sync1(X) is the average (global average) of Correlation for all the Nt combinations and for all the Ns number of stimuli.

% Calculation of the array Sync2(C, X) for each EEG channel

I = 0 ; U = 0;

for Ax = 1 : (NC - 1)

for Bx = (Ax + 1) : NC

I = I + 1 ; % counter of all the combinations of the channels

for U = 1 : NC

if (U == Ax) || (U == Bx)

for X = 1 : N1

Sync2(U, X) = Sync2(U, X) + abs(R(I, X) - Bs(I)) ;

end

end

end

end

end

for C = 1 : NC

for X = 1 : N1

Sync2(C, X) = Sync2(C, X) / (NC-1) ;

end

end

% Now the array Sync2(C, X) is the Correlation for each channel.

% Each channel is the average of (NC-1) data.

% Simple example of the ClassicERP.m calculation

% NC are the EEG channels, N1 the number of samples time-locked

% if N1 = 452 as in the W(C, X, J) array, a final cutting of two tails of length L = 34 should be done

% like in the GW6.m elaboration.

Ev= zeros(NC, N1); % Ev(C, X) is the array of classic ERP for each EEG channel

for J = 1 : Ns % for all the stimuli

for C = 1 : NC %for all the channels

for X = 1 : N1

Ev(C, X) = Ev(C, X) + W(C, X, J) ;

end

end

end

for C = 1 : NC

for X = 1 : N1

Ev(C, X) = Ev(C, X) / Ns ;

end

end

% Now the array Ev(C, X) is the classic ERP .
